# Resource Supply Overrides Temperature as a Controlling Factor of Marine Phytoplankton Growth

**DOI:** 10.1371/journal.pone.0099312

**Published:** 2014-06-12

**Authors:** Emilio Marañón, Pedro Cermeño, María Huete-Ortega, Daffne C. López-Sandoval, Beatriz Mouriño-Carballido, Tamara Rodríguez-Ramos

**Affiliations:** 1 Departamento de Ecología y Biología Animal, Universidad de Vigo, Vigo, Spain; 2 Instituto de Ciencias del Mar, Consejo Superior de Investigaciones Científicas, Passeig Maritim de la Barceloneta 37–49, Barcelona, Spain; 3 Department of Chemical and Biological Engineering, The University of Sheffield, Sheffield, United Kingdom; Stazione Zoologica Anton Dohrn, Naples, Italy

## Abstract

The universal temperature dependence of metabolic rates has been used to predict how ocean biology will respond to ocean warming. Determining the temperature sensitivity of phytoplankton metabolism and growth is of special importance because this group of organisms is responsible for nearly half of global primary production, sustains most marine food webs, and contributes to regulate the exchange of CO_2_ between the ocean and the atmosphere. Phytoplankton growth rates increase with temperature under optimal growth conditions in the laboratory, but it is unclear whether the same degree of temperature dependence exists in nature, where resources are often limiting. Here we use concurrent measurements of phytoplankton biomass and carbon fixation rates in polar, temperate and tropical regions to determine the role of temperature and resource supply in controlling the large-scale variability of *in situ* metabolic rates. We identify a biogeographic pattern in phytoplankton metabolic rates, which increase from the oligotrophic subtropical gyres to temperate regions and then coastal waters. Variability in phytoplankton growth is driven by changes in resource supply and appears to be independent of seawater temperature. The lack of temperature sensitivity of realized phytoplankton growth is consistent with the limited applicability of Arrhenius enzymatic kinetics when substrate concentrations are low. Our results suggest that, due to widespread resource limitation in the ocean, the direct effect of sea surface warming upon phytoplankton growth and productivity may be smaller than anticipated.

## Introduction

Temperature governs the metabolism of all organisms. Within favourable thermal ranges, there exists a positive, often exponential, increase in metabolic rate as temperature rises [1], [2]. On-going and predicted ocean warming [3] prompts the question of how phytoplankton photosynthetic activity and growth may respond to increasing sea surface temperatures [4]–[6]. This response will be relevant for the functioning of the Earth system as a whole, because phytoplankton sustain most marine food webs and, being responsible for nearly half of the global primary production [7], contribute to control the exchange of CO_2_ and other radiatively active gases between the ocean and the atmosphere [8], [9].

Laboratory work with cultures growing under optimal conditions has shown that the maximum growth rates of phytoplankton, and thus their maximum rate of biomass-specific production, increase exponentially with temperature with a *Q*_10_ of approximately 2 within the tolerable temperature range [10]–[13]. However, it is not clear whether phytoplankton assemblages in nature show the same degree of temperature dependence in their *realized* rates of metabolic activity and growth. An analysis of *in vitro* oxygen evolution data across the global ocean suggests that phytoplankton photosynthesis does increase with temperature and, furthermore, that the degree of temperature dependence is similar to that predicted by the metabolic theory of ecology [14]. Similarly, an eco-evolutionary model predicts that phytoplankton living in tropical regions sustain faster growth rates than their counterparts living in temperate and polar regions [6]. These studies, however, did not consider the role of resource supply, which is included in applications of the metabolic theory of ecology [1], [15] and limits phytoplankton production and growth in most of the open ocean [16]. Nutrient limitation has been shown to reduce the temperature sensitivity of carbon fixation by phytoplankton [17]–[19]. If resource limitation attenuates the temperature dependence of metabolic rates, different regions where phytoplankton experience different degrees of resource limitation may show contrasting productivity responses to warming, and models that use laboratory-based values of *Q*_10_ are likely to overestimate the direct effects of temperature upon algal growth.

Understanding the variability and controlling factors of phytoplankton growth rates is key to predict the response of ocean biology to external forcings such as climate variability [20]. However, in spite of decades of observation [10], [20]–[23], a general picture of phytoplankton growth variation over broad spatial scales in the ocean remains elusive. Here, we use concurrent data of phytoplankton carbon biomass and photosynthetic carbon fixation, determined in coastal and open-ocean waters of polar, temperate and tropical biomes, to investigate the large-scale variability of phytoplankton metabolic rates, represented by the biomass-specific rate of carbon fixation [10], [24]. Our analysis allows us to characterize biogeographic patterns in phytoplankton growth and to assess the relative role of temperature and resource availability in the control of marine primary productivity.

## Materials and Methods

### Data acquisition

We compiled data on phytoplankton carbon biomass concentration and primary production rate that were determined concurrently in surface assemblages of coastal and open-ocean regions. Coastal observations (number of sampling visits, *n* = 26) were conducted throughout the year at a central station (depth = 40 m) in the Ría de Vigo (NW Iberian Peninsula), a highly productive embayment subject to frequent upwelling events, particularly in spring and summer [25], [26]. Open-ocean stations (*n* = 38) were visited in April-May 1996 and September-October 1996 along the Atlantic Meridional Transect, which crosses temperate, subtropical and tropical regions in the north and south Atlantic Ocean [27]. The data from the AMT stations were grouped according to their latitude into north temperate (35–49°N), south temperate (35–48°S), north oligotrophic (20–31°N), south oligotrophic (10–34°S) and equatorial and Mauritanian upwelling (5°S–20°N). Additional data (*n* = 8) were obtained from the Soiree (Southern Ocean Iron Release Experiment) study, conducted in open-ocean, polar waters of the Australasian-Pacific Southern Ocean [28]–[30]. To minimise the effects of vertical variability in irradiance upon phytoplankton photosynthetic activity, the present analysis uses only data from surface samples (0–5 m in coastal waters, 0–20 m in open-ocean waters).

### Phytoplankton biomass and production

Phytoplankton carbon biomass was estimated from measurements on fixed samples of cell abundance and biovolume, determined with flow cytometry for the picophytoplankton and with an inverted microscope for the nano- and micro-phytoplankton, following protocols that are detailed elsewhere [26], [27], [30]. Biovolume data were transformed into carbon biomass by applying appropriate conversion factors. Primary production rate was determined with the ^14^C-uptake technique using on-deck incubations which simulated *in situ* temperature and irradiance conditions [26], [27], [29]. Phytoplankton biomass turnover rates (d^−1^), equivalent to intrinsic growth rates [10], [24], were calculated by dividing the daily carbon fixation rate by phytoplankton carbon biomass.

### Hydrography data

Vertical profiles of temperature and salinity were obtained with a CTD probe attached to a rosette sampling system. Density was calculated from temperature and salinity by using the standard UNESCO equation. The concentration of dissolved nitrate in the euphotic layer was determined by segmented-flow analysis using an automatic analyser and standard colorimetric protocols [26], [27], [31]. The detection limit of this analysis (0.05 *µ*mol L^−1^) was sufficient to measure nitrate concentration at the base of the euphotic layer, which was always >0.2 *µ*mol L^−1^. The depth of the euphotic layer was determined from vertical profiles of irradiance obtained with a PAR Li-Cor sensor.

### Index of resource supply

We computed a resource supply index (*RSI*) that takes into account the concentration of nitrate at the base of the euphotic zone (*NO_3_*_[1%PAR]_), the density difference between the surface and the base of the euphotic zone (Δσ*_t_*), the depth of the euphotic zone, defined as the 1% PAR level (1%*PARz*), and the depth of the upper mixed layer (*UMLz*), defined as the first depth at which σ*_t_* is 0.125 units higher than the surface value [32]:
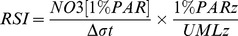
(1)


The first term in Eq. 1 reflects the fact that nutrient transport to the upper layer increases with increasing nutrient concentration below, but also that this transport becomes progressively more limited as vertical stratification intensifies. The second term in Eq. 1 serves to differentiate conditions in which phytoplankton are confined to a shallow upper mixed layer, relative to the depth of the euphotic zone, and thus experience relatively high average irradiances, from conditions in which intense mixing results in mixed layers whose depth equals or exceeds the penetration of irradiance into the water column, potentially resulting in light limitation of phytoplankton growth. Our index of resource supply is not intended to provide an accurate estimate of local-scale resource availability, but is used here to depict broad differences between regions with widely contrasting hydrographic regimes. *RSI* was not calculated for the stations sampled in the Soiree study, where, in addition to macronutrients and irradiance, iron was a limiting factor for phytoplankton production and growth [28].

### Statistical analyses

Pearson's *r* was calculated to assess the existence of linear dependence between variables. We applied reduced major-axis regression to determine the linear relationship between log_10_-transformed variables. Ninety-five percent confidence intervals for the regression parameters were calculated by bootstrapping over cases (2,000 repetitions). The non-parametric Mann-Whitney *U* test was used to compare means between groups of stations.

## Results

### Phytoplankton biomass and production

**Figure 1:**
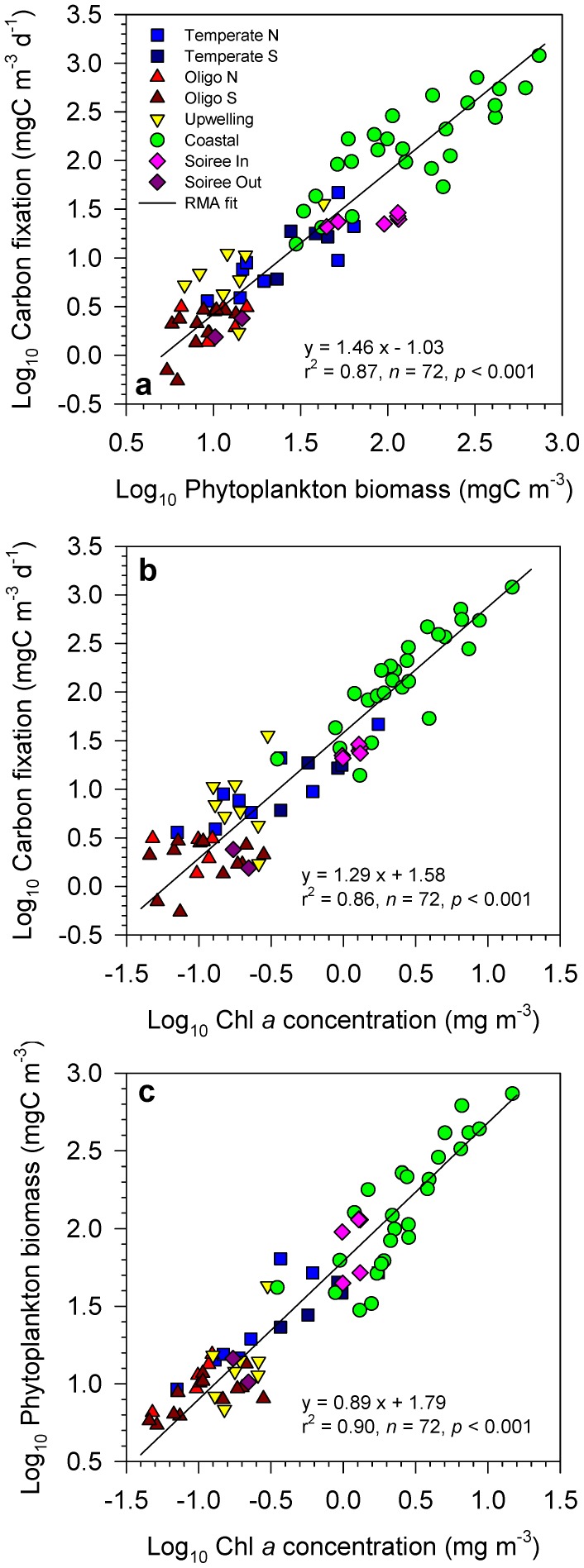
Phytoplankton chlorophyll *a*, biomass and production. Scatterplots showing the log-log relationship between (a) primary production and phytoplankton carbon biomass, (b) primary production and chlorophyll *a* concentration and (c) phytoplankton carbon biomass and chlorophyll *a* concentration. Different symbols indicate the sampling region as defined in the Methods section. ‘Soiree In’ and ‘Soiree Out’ refer to stations located inside and outside, respectively, the iron-fertilized patch during the Soiree study. Data were fitted to a linear model using reduced major axis regression.

Across all studied regions, which showed widely contrasting hydrographic properties (Table 1), carbon fixation rates covaried closely with phytoplankton standing stocks, represented either by carbon biomass (Fig. 1a) or by chlorophyll *a* concentration (chl *a*) (Fig. 1b). The range of variability in phytoplankton production rates was >3 orders of magnitude, compared with approximately 2 orders of magnitude for phytoplankton carbon biomass and chl *a*. The key feature of these relationships is that in both cases the slope of the fitted line is significantly higher than 1, taking a value of 1.46 (95% confidence interval: 1.35, 1.58) in the production-carbon biomass regression and 1.29 (1.19, 1.39) in the production-chl *a* regression. This result implies that, as phytoplankton standing stocks increase, primary production rates increase at a faster rate. From oligotrophic regions to coastal waters, mean phytoplankton chl *a* and biomass increased by a factor of 20–30, whereas primary production increased by a factor of >100 (Table 2).

**Table 1:** Physical and chemical properties of the studied regions.

Location	Temp.	*NO_3_*_[1%PAR]_	Δσ*_t_*	1%*PARz*	*UMLz*	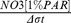	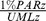
Temperate N (*n* = 8)	15.9±3.5	6.7±1.6	0.6±0.5	68±23	64±35	28±29	1.4±0.8
Temperate S (*n* = 4)	13.4±4.5	9.8±4.2	0.4±0.2	57±22	50±15	33±26	1.2±0.3
Oligotrophic N (*n* = 5)	22.3±2.3	1.9±1.5	0.8±0.5	115±14	47±13	2.8±1.5	2.6±0.8
Oligotrophic S (*n* = 13)	24.4±2.9	0.9±1.2	0.9±0.6	126±24	59±22	1.1±1.5	2.6±1.4
Upwelling (*n* = 8)	27.3±1.4	14.0±2.1	3.2±0.6	83±13	29±12	4.4±0.9	3.3±1.6
Coastal (*n* = 26)	15.3±2.0	6.8±3.2	0.8±0.6	20±4	7±4	11.5±7.6	3.9±2.2
Soiree In (*n* = 6)	2.8±0.1	23.2±1.1	0.02±0.01	47±1	72±3	-	-
Soiree Out (*n* = 2)	2.7±0.0	25.4±0.4	0.23±0.02	74±8	69±2	-	-

Mean (± standard deviation) values of sea surface temperature (°C, Temp.), nitrate concentration at the base of the euphotic layer (*NO_3_*_[1%PAR]_, *µ*mol L^−1^), density difference between the surface and the base of the euphotic layer (Δσ*_t_*, kg m^−3^), euphotic layer depth (1%*PARz*, m), and upper mixed layer depth (*UMLz*, m) for each location. For the regions where the resource supply index was calculated, the mean values of *NO_3_*_[1%PAR]_ divided by Δσ*_t_* and 1%*PARz* divided by *UMLz* are also given. *n* is the number of measurements conducted at each location. Nitrate concentration data for the Soiree study correspond to 40 m depth [31].

**Table 2:** Phytoplankton properties at the studied regions.

Location	Chl *a*	Phyto C	C:Chl *a*	P
Temperate N (*n* = 8)	0.44±0.56	30±22	99±42	13±15
Temperate S (*n* = 4)	0.71±0.29	34±10	50±10	15±6
Temperate S (*n* = 4)	0.71±0.29	34±10	50±10	15±6
Oligotrophic N (*n* = 5)	0.10±0.03	11±3	117±15	2.5±0.8
Oligotrophic S (*n* = 13)	0.13±0.07	9±2	83±31	2.1±0.8
Upwelling (*n* = 8)	0.20±0.07	16±11	77±36	10±11
Coastal (*n* = 26)	3.5±3.2	202±187	58±28	248±271
Soiree In (*n* = 6)	1.19±0.16	89±33	75±25	25±3
Soiree Out (*n* = 2)	0.20±0.03	12±3	65±27	2.0±0.6

Mean (± standard deviation) surface values of chlorophyll *a* concentration (Chl *a*, mg m^−3^), phytoplankton carbon biomass (Phyto C, mgC m^−3^), phytoplankton carbon to chlorophyll *a* ratio (C:Chl *a*), and rate of primary production (P, mgC m^−3^ d^−1^) in each location. *n* is the number of stations visited at each location.

The relationship between chl *a* and phytoplankton carbon (Fig. 1c) was remarkably robust, considering the uncertainties involved in estimating biomass from biovolume and the fact that the samples were analysed by several independent laboratories. The slope of the log-log regression between phytoplankton carbon and chl *a* (0.89; 95% CI: 0.83, 0.94) was significantly lower than 1, indicating that the C:Chl *a* ratio tends to decrease as phytoplankton standing stocks increase from open-ocean oligotrophic to open-ocean temperate and then coastal waters (Table 2). This pattern likely reflects the fact that in temperate and coastal waters phytoplankton are acclimated to lower mean irradiances, which results in increased chl *a* cellular content.

### Temperature and phytoplankton growth

**Figure 2:**
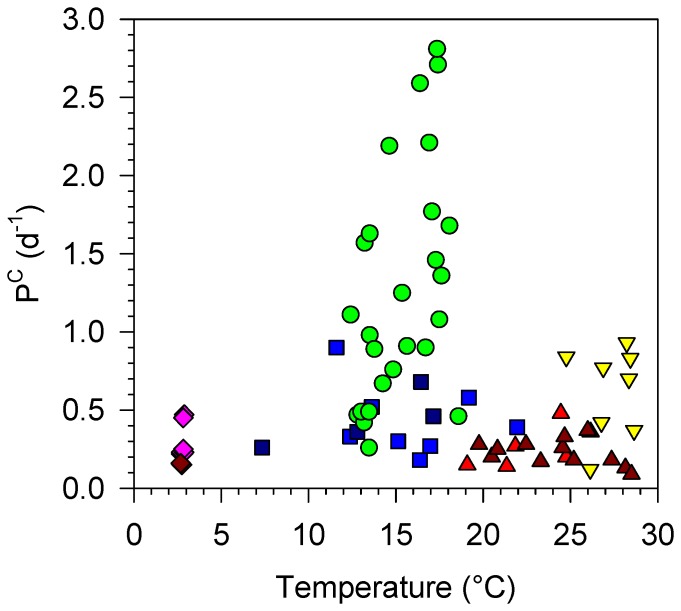
Temperature and phytoplankton growth. Carbon-specific production rate (P^C^) plotted against temperature for all stations. Symbols as in Fig. 2.

We found no overall relationship between phytoplankton growth rate, represented by the biomass turnover rate (P^C^), and temperature (Pearson's *r* = −0.045, *p*>0.3, *n* = 72) (Fig. 2). The highest P^C^ values (>1 d^−1^) were measured in coastal waters, despite the fact that their temperature was 10–15°C lower than that of subtropical and tropical oligotrophic regions (Table 1). Some of the coldest and the warmest waters analysed had similarly low P^C^ values (<0.4 d^−1^). During the Soiree study, P^C^ inside the iron-fertilised patch took higher values than in surrounding waters with the same temperature. The stations within the Atlantic upwelling region tended to have higher P^C^ values than oligotrophic stations with similar temperatures.

### Resource supply and phytoplankton growth

**Figure 3:**
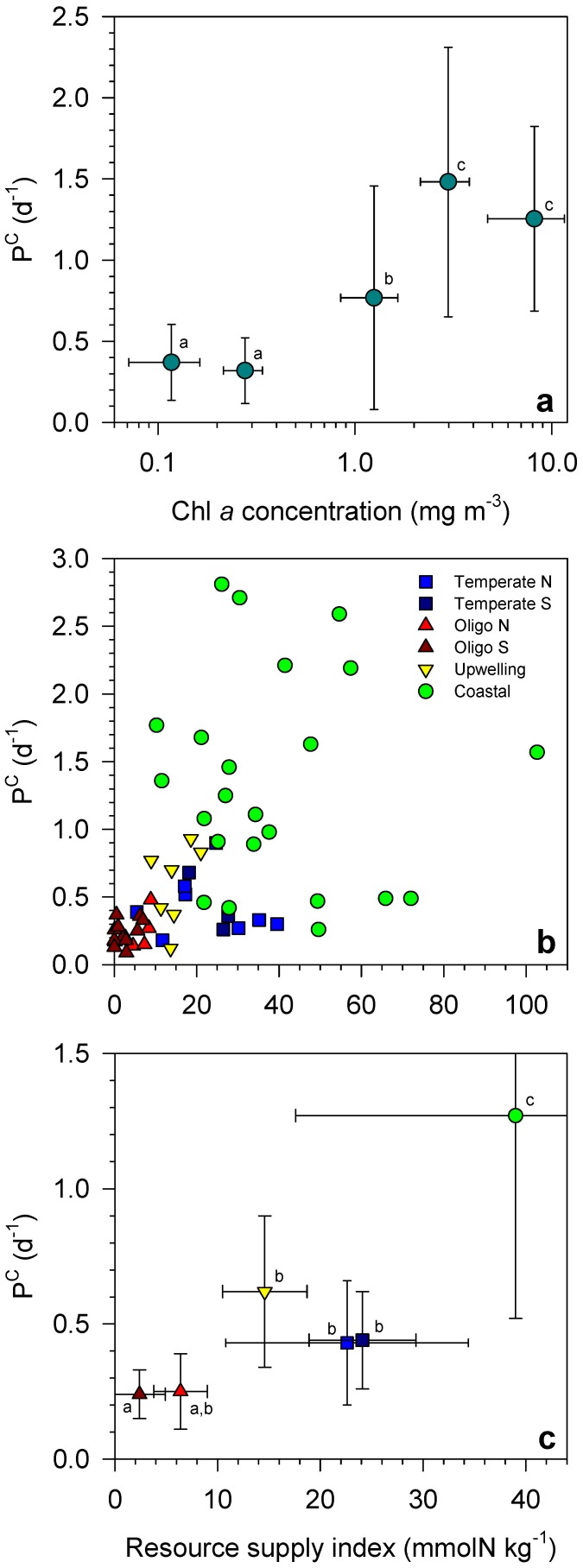
Resources and phytoplankton growth. Carbon-specific production rate (P^C^) as a function of (a) chlorophyll *a* concentration (chl *a*) and (b, c) resource supply index (*RSI*). P^C^ data in (a) were grouped into the following chl *a* ranges: 0–0.2, 0.2–0.5, 0.5–2, 2–5 and >5 mg m^−3^. Bars indicate ±1 standard deviation. Data groups labelled with different letters in (a) and (c) have significantly different P^C^ values (Mann-Whitney *U* test, *p*<0.05). The units of *RSI* derive from the division of nitrate concentration by seawater density. Note that *RSI* is not a nutrient concentration, but reflects both nutrient and light availability (see Material and Methods for details).

P^C^ increased with chl *a* (Fig. 3a), suggesting that an enhanced supply of resources, necessary to sustain larger standing stocks, leads to faster phytoplankton growth. Groups of samples with mean chl *a* above 2 mg m^−3^ had mean P^C^ values higher than 1.2 d^−1^, whereas samples with mean chl *a* below 0.2 mg m^−3^ showed P^C^ values below 0.4 d^−1^. These differences were statistically significant (Mann-Whitney's *U* test, *p*<0.05). The role of resource supply in controlling phytoplankton biomass turnover was confirmed by the positive correlation between the resource supply index (*RSI*) and P^C^ (Pearson's r = 0.45, *p*<0.001, *n* = 59; Fig. 3b). *RSI* was calculated as a function of nitrate concentration at the base of the euphotic layer, the density difference between sea surface and the base of the euphotic layer, and the depths of the upper mixed layer and the euphotic zone (see Material and Methods). *RSI* varied widely, reflecting large across-system differences in nutrient concentration and water column stratification (Table 1). The oligotrophic regions showed low *RSI* values (<10 mmolN kg^−1^) (Fig. 3c), mainly due to their low nitrate concentration at the base of the euphotic layer (Table 1). Higher nitrate concentration in the temperate regions resulted in *RSI* values above 20 mmolN kg^−1^. The highest *RSI* values were determined in the Ría de Vigo, where high nitrate concentrations at the base of the euphotic layer co-occur with upper mixed layers that are shallow relative to the depth of the euphotic layer (Table 1). Despite having similar nitrate concentration at the base of the euphotic zone, the temperate regions had lower *RSI* values than the coastal waters (Fig. 3c), because the former were characterized by deeper mixed layers relative to the depth of the euphotic zone. Finally, *RSI* was lower in the upwelling region than in the coastal waters of Ría de Vigo, even though deep nitrate concentration was higher in the former, because of the strong thermal stratification, associated with the upwelling, that resulted in a relatively large density gradient (Table 1).

There was an overall positive relationship between P^C^ and *RSI* across all studied regions (Fig. 3c). The open-ocean temperate and upwelling regions had faster biomass turnover rates than the oligotrophic regions and in turn the coastal waters showed faster growth rates than all other regions. The oligotrophic stations of the south Atlantic gyre, characterized by the lowest *RSI* values, showed mean P^C^ values that were significantly lower than those determined in the temperate and upwelling open-ocean regions and in the coastal waters (Mann-Whitney's *U* test, *p*<0.05). The differences in P^C^ between coastal waters and all other regions were also statistically significant (Mann-Whitney's *U* test, *p*<0.05).

**Figure 4:**
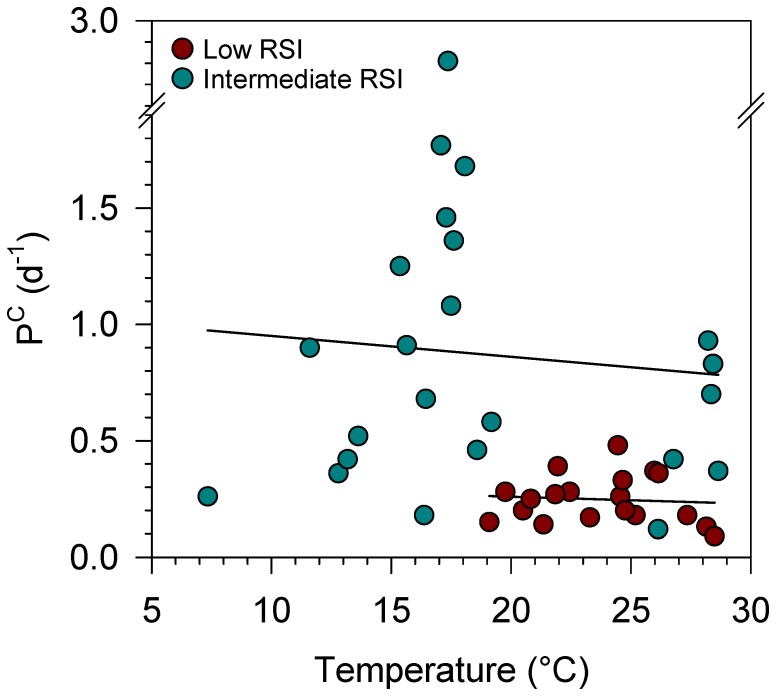
Temperature and phytoplankton growth in samples with different resource availability. Carbon-specific production rate (P^C^) is plotted against temperature for oligotrophic stations with low resource supply index (*RSI*<10 mmolN kg^−1^) and temperate, upwelling and coastal stations with intermediate *RSI* (10–20 mmolN kg^−1^). Both linear regression fits were non-significant (*p*>0.6).

The relationship between temperature and phytoplankton growth rate could be masked due to the fact that most of the variability in P^C^ was due to changes in resource supply. To test this possibility, we plotted temperature against P^C^ in groups of stations which had similar resource supply conditions, as indicated by their *RSI* values, and covered a temperature range of at least 10°C (Fig. 4). In both low-*RSI* and intermediate-*RSI* stations, we found no significant relationship between temperature and P^C^.

## Discussion

Our results suggest that the large-scale variability of realized mass-specific carbon fixation rates, and hence intrinsic growth rates [33], [34], of marine phytoplankton is not controlled by seawater temperature, but depends primarily on resource supply. Given that under resource-saturated conditions phytoplankton growth does show a clear temperature dependence [10], [13], [35], our observations imply that temperature and resource limitation effects in phytoplankton are not independent. We hypothesise that the lack of effect of temperature upon phytoplankton metabolic rate under resource-limited conditions arises because the applicability of Arrhenius kinetics is limited when substrate concentration are low [36]. The maximum reaction rate (*V*_max_) of an enzyme increases with temperature, up to an optimal temperature, due to higher substrate kinetic energy and enhanced collision rate between reactants. However, an increase in temperature also causes reduced ligand binding ability [37], and as a result the Michaelis-Menten constant (*K*_m_) of most enzymes also increases with temperature [38]–[40]. Thus, the temperature sensitivities of *V*_max_ and *K*_m_ can neutralize each other and give way to *Q*_10_ values near 1 (absence of temperature-dependence) when substrate concentrations are around or below *K*_m_ [36], [41]. Our hypothesis is consistent with the analysis of Raven and Geider [12], who concluded that nutrient limitation leads to a smaller temperature dependence of growth, compared with nutrient-saturated conditions. Moreover, experimental studies with natural phytoplankton assemblages have shown that under nutrient limitation the temperature dependence of photosynthetic rate is reduced [17] or disappears altogether [18].

The lack of relationship between temperature and phytoplankton growth rate in our data may seem at odds with the results of some experimental studies in open-ocean waters which have shown that phytoplankton metabolism responds to temperature changes [42], [43]. However, these manipulation experiments reflect short-term, transient responses in phytoplankton physiology and therefore do not capture the large acclimation potential of phytoplankton [44]. The present results are also in contrast to the findings of Regaudie-de-Gioux and Duarte [14], who concluded that there is a strong temperature dependence of phytoplankton photosynthesis in the sea. The discrepancy is likely to result from the fact that in their analysis these authors pooled together data obtained throughout the euphotic zone, thus introducing irradiance as a covarying factor. Samples near the base of the euphotic zone, where temperatures are colder than at the surface, will necessarily show lower rates of photosynthesis, not as a result of the lower temperature but as a result of lower irradiance. In addition, photosynthetic rates in the study by Regaudie-de-Gioux and Duarte were normalised by chlorophyll *a* instead of carbon biomass. However, it is well-established that the cellular content of chlorophyll *a* increases with decreasing irradiance and increasing nutrient availability [45]–[47]. Therefore, normalising metabolic rates by chlorophyll *a* results in biased patterns of temperature dependence whenever there is covariation between temperature, nutrient availability and irradiance.

Our observations depict a consistent, broad-scale pattern in phytoplankton growth rates, which increase from oligotrophic gyres to temperate, open-ocean regions and then to coastal, productive waters. This resource-driven pattern is associated with changes in community structure [26], [27], as oligotrophic waters are dominated by small cells (picocyanobacteria and picoeukaryotes) with relatively low biomass turnover rates, whereas the most productive waters are dominated by fast-growing diatoms. Warm temperatures and a tight coupling between zooplankton-mediated nutrient regeneration and phytoplankton uptake could, in principle, result in an absence of nutrient limitation and near-maximal phytoplankton growth rates in the nutrient-impoverished subtropical gyres [20], [21], [23]. This possibility, however, contrasts with the experimental evidence showing physiological stress of the dominant picophotoautotrophs in the oligotrophic Atlantic, which is alleviated upon addition of nitrogen [16], [48], [49]. Our results support the existence of nutrient limitation of both standing stocks and growth rates in the oligotrophic gyres [50], i.e. a situation in which both Liebig's and Blackman's types of limitation apply [16], [51]. This limitation appears to be less acute in temperate open-ocean regions, which are subject to seasonal nutrient injections through vertical mixing, and in the upwelling open-ocean region, where upward water motion enhances the nutrient supply to the euphotic zone. In the coastal, productive waters of Ría de Vigo, high resource supply results in growth rates that are, on average, similar to the maximum growth rates expected given the *in situ* temperatures. These resource-driven biogeographic patterns imply a predominantly bottom-up control of phytoplankton growth in the ocean.

A previous study [52] has shown that phytoplankton growth rates in coastal, nutrient-rich waters show a degree of temperature dependence comparable to that observed in laboratory cultures, which confirms that temperature sets an upper limit to the maximum growth rates that phytoplankton can achieve under resource-saturated conditions. In addition, nutrient stoichiometry and pigment content have been shown to be temperature-dependent, reflecting intracellular changes in the abundance of macromolecules involved in light harvesting, photochemistry and biosynthesis [12], [53], [54]. Furthermore, inter-species differences in thermal tolerance ranges and optimal temperature for growth [55] contribute to explain changes in species distribution, community structure and associated ecosystem functioning over multiple temporal and spatial scales [56]–[59]. Nevertheless, as far as the large-scale variability in phytoplankton production and growth rates is concerned, temperature seems to be unimportant whereas resource supply plays a crucial role. This result may have important consequences for the modelling of ocean ecosystem responses to climate change. Biogeochemical models typically estimate phytoplankton growth rates as a multiplicative function of nutrient concentration, irradiance, and temperature-dependent maximum growth rate [5], [60], [61]. This formulation assumes that temperature effects on growth are independent of resource supply status, so that for a given condition of resource availability, an increase in temperature will necessarily result in faster phytoplankton growth. In contrast, our observations indicate that resource limitation drastically reduces the temperature dependence of phytoplankton metabolism. Similarly, the growth [62] and extracellular enzymatic activities [63] of natural marine bacterial assemblages show only a very limited response to temperature when resources are in low supply. Hence, the sensitivity of microbial metabolic rates to sea surface warming, particularly in open-ocean, nutrient-limited regions, may be smaller than anticipated [4], [14], [64], [65].
